# Evaluation and Management of Knee Dislocations in Low-income Settings: Evidence-based Strategies and Practical Considerations

**DOI:** 10.5435/JAAOSGlobal-D-25-00006

**Published:** 2025-10-14

**Authors:** Camilo Partezani Helito, Carlos Eduardo da Silveira Franciozi, Andre Giardino Moreira da Silva, Diego Ariel de Lima

**Affiliations:** From the USP - Universidade de São Paulo, Brazil (Dr. Helito and Dr. Moreira da Silva); UNIFESP - Universidade Federal de São Paulo, Brazil (Dr. Franciozi); and UFERSA - Universidade Federal Rural do Semi-Árido, Brazil (Dr. Ariel de Lima).

## Abstract

Knee dislocations are rare but severe injuries, often resulting from high-energy trauma and commonly associated with vascular and neurologic damage. In low-income settings, the evaluation and management of these injuries are especially challenging because of limited access to advanced imaging, surgical implants, and structured rehabilitation services. This review aims to provide a practical, evidence-based framework for managing knee dislocations in resource-constrained environments. A focused literature review was conducted on studies published in the past decade that address diagnosis, treatment strategies, and system-level barriers in underserved regions. Key topics include simplified diagnostic approaches using clinical evaluation and basic imaging, early detection of vascular and nerve injuries without advanced tools, and cost-effective surgical techniques. These include the use of autografts, external fixators, and implantless fixation methods such as press-fit and bone bridges, as well as the use of the same tunnels and fixation devices for more than one ligament. Rehabilitation strategies are also adapted, with emphasis on simplified protocols that consider social and logistical barriers to follow-up. Despite notable limitations, appropriate prioritization, creative use of available materials, and staged reconstructions can ensure acceptable outcomes. This article offers orthopaedic surgeons working in low-resource environments practical guidance to maintain standards of care through adaptable, cost-conscious decision-making.

Knee dislocation is a rare but severe injury characterized by complete disruption of the tibiofemoral articulation. It is frequently associated with damage to critical neurovascular structures, particularly the popliteus artery and the peroneal nerve, making timely diagnosis and intervention essential to prevent limb loss or long-term disability.^1,2^

In high-income settings, access to advanced imaging, multidisciplinary trauma teams, and a wide range of surgical implants allows for standardized protocols and improved outcomes. However, in low-income and resource-constrained environments, these injuries present unique challenges. Limited availability of imaging modalities, operating room infrastructure, and postoperative rehabilitation often leads to delayed treatment and suboptimal results.^3,4^ In many low-resource regions, high-energy trauma from motorcycle crashes and pedestrian-vehicle collisions is a leading cause of knee dislocations, disproportionately affecting young, working-age individuals.^5^

Although comprehensive reviews on multiligament knee injuries (MLKIs) exist, few address the specific needs and constraints of surgeons operating in low-resource settings. This article aims to provide an evidence-based framework for evaluating and managing knee dislocations in such environments. It focuses on diagnostic strategies adapted to basic tools, prioritization of vascular and neurologic injuries, cost-conscious surgical techniques including implantless options, and simplified rehabilitation protocols suitable for vulnerable populations.

By contextualizing the management of knee dislocations within the realities of underserved regions, this review seeks to fill a relevant gap in the orthopaedic literature and support clinicians practicing in global health scenarios.

## Review Criteria/Methods

A targeted literature search was conducted using PubMed/MEDLINE, Cochrane Library, and Scopus to identify relevant articles published between January 2013 and May 2025. Search terms included combinations of “knee dislocation,” “multiligament knee injury,” “low-resource settings,” “developing countries,” “vascular injury,” “implantless fixation,” and “cost-effective orthopedics.”

Studies were eligible for inclusion if they focused on the evaluation, diagnosis, surgical treatment, or rehabilitation of knee dislocations in adult populations, particularly in low- or middle-income countries or resource-limited environments. Both traumatic and spontaneous knee dislocations were considered, but the emphasis was placed on high-energy trauma and multiligament injuries.

Eligible articles included clinical trials, observational studies, systematic and narrative reviews, case series, case reports, expert consensus documents, and global surveys. Articles not available in English, studies focused exclusively on pediatric or oncologic populations, and those unrelated to constrained-resource environments were excluded.

The literature was critically reviewed to extract practical strategies and evidence applicable to underserved contexts. The authors' clinical experience in low-income public healthcare systems also informed the interpretation and selection of relevant content.

## Epidemiology and Global Burden

Knee dislocations are relatively rare injuries, accounting for less than 0.02% of all orthopaedic trauma admissions in high-income countries. However, in low- and middle-income settings, the true incidence may be markedly underestimated due to underreporting, limited diagnostic capabilities, and variations in trauma registries.^6,7^

In high-resource regions, sports-related trauma is the predominant cause of knee dislocations, often presenting as low- to moderate-energy injuries with isolated ligamentous compromise. By contrast, in low-income countries, most knee dislocations result from high-energy mechanisms such as motorcycle accidents, pedestrian-vehicle collisions, or falls from height—particularly in densely populated urban centers.^2,3^ These injuries typically involve extensive multiligament disruption and a higher risk of associated vascular and neurologic compromise.

A study by Venter et al,^4^ based on a global survey of orthopaedic surgeons, highlighted that delayed diagnosis and treatment are more prevalent in low-resource settings, often due to a lack of access to emergent imaging and specialist care. In addition, Spiegel et al^5^ emphasized the burden of musculoskeletal trauma in low-income countries as a leading contributor to long-term disability among working-age adults.

Risk factors for knee dislocation in these environments include high rates of road traffic accidents, poor enforcement of safety regulations (eg, helmet and seat belt use), limited emergency transport systems, and delayed hospital presentation. These factors not only influence the severity of injury but also affect the timing and quality of treatment.^7-9^

Addressing the global burden of knee dislocations requires a context-sensitive understanding of these epidemiologic patterns to guide prevention, early recognition, and resource-appropriate management strategies.

## Initial Evaluation and Diagnosis in Resource-limited Settings

The initial evaluation of a suspected knee dislocation must begin with a thorough clinical assessment, given the high risk of associated vascular and neurologic injuries. In resource-constrained settings, where access to advanced imaging may be delayed or unavailable, clinical vigilance and systematic examination are essential.

A visibly deformed, unstable, or shortened limb should raise immediate suspicion for dislocation, even in cases where spontaneous reduction has occurred before evaluation. Palpation of distal pulses—particularly the dorsalis pedis and posterior tibial arteries—is mandatory and should be compared with the contralateral limb. Capillary refill, skin temperature, and limb color provide additional clues to vascular status. The ankle-brachial index (ABI) remains a low-cost, reliable tool: Values <0.9 suggest potential arterial injury and warrant additional vascular investigation.^1,10^

Neurologic assessment should include motor and sensory testing of the peroneal and tibial nerves, with special attention to foot dorsiflexion and plantarflexion, as well as light touch sensation over the dorsum and sole of the foot. Ultrasound may also be employed to characterize the pattern of peroneal nerve injury, thereby aiding surgical planning, especially in cases of complete disruption (neurotmesis). Peroneal nerve palsy, either transient or permanent, is reported in up to 40% of high-energy knee dislocations.^11^

In the absence of overt ischemia, Doppler ultrasonography is a valuable first-line tool in low-resource environments. It is portable, widely available, and allows dynamic assessment of arterial flow. If Doppler findings are abnormal or inconclusive, CT angiography (CTA) is preferred—when available—due to its high sensitivity and specificity.^12^ Plain radiographs (anterior-posterior and lateral) are essential to confirm joint alignment, assess for fractures, and exclude spontaneous relocation. When accessible, point-of-care ultrasonography can also assist in identifying hemarthrosis or posterior capsule disruptions.^13^

The challenge in these settings is to balance urgency with pragmatism: Clinical judgment must drive decision-making when technology is lacking. Early recognition and documentation of vascular and neurologic status are crucial to guide timely referral or intervention.

## Vascular and Neurologic Injury Management

Vascular injury remains the most critical and time-sensitive complication of knee dislocation. The popliteus artery is particularly vulnerable due to its fixation in the adductor hiatus and behind the knee joint. Missed or delayed diagnosis can lead to irreversible ischemia, limb loss, and even mortality. In resource-limited environments, early clinical recognition and streamlined referral protocols are essential to optimize outcomes.^1,14^

The initial vascular assessment should include pulse palpation, limb temperature, capillary refill, and measurement of the ABI. ABI values <0.9 after reduction warrant additional investigation. When advanced imaging is unavailable, portable Doppler ultrasonography serves as a reliable tool to detect gross flow abnormalities and should be incorporated into emergency protocols.^10^

Revascularization is indicated in cases of absent distal pulses or confirmed arterial transection, preferably within 6 to 8 hours of injury. In settings without in-house vascular surgery, rapid referral to a higher-level center must be prioritized. When available, temporary intraluminal vascular shunts may be used for limb salvage before definitive repair.^15^ Simultaneous stabilization with an external fixator can facilitate transport and vascular repair.

Nerve injury, particularly peroneal nerve palsy, occurs in up to 40% of high-energy knee dislocations, often presenting with foot drop and sensory loss. In the acute phase, observation is recommended unless there is clear evidence of nerve transection or open injury. If no improvement is observed within two months, surgical decompression should be considered. When a lateral procedure is required for fracture fixation or ligament insufficiency, nerve decompression is mandatory in cases of peroneal nerve palsy without neurotmesis.^16-18^

Protective bracing treatment (AFO) and early physical therapy are essential to prevent contractures and secondary deformities. Tendon transfer may be considered in cases of persistent deficit after 6 to 12 months, depending on local surgical expertise.^1,14^

Despite limited tools, timely recognition, serial clinical monitoring, surgical decompression after two months without improvement, and coordinated care pathways can markedly improve neurovascular outcomes in patients with knee dislocations.

## Acute Stabilization and Initial Management

Initial management of knee dislocation begins with prompt recognition and immediate reduction of the dislocated joint. Closed reduction under conscious sedation or local anesthesia should be done as soon as possible, especially in cases with neurovascular compromise. Gentle longitudinal traction followed by directed manipulation usually restores alignment. Postreduction, distal pulses must be reassessed and compared with prereduction status.^11,19^

Temporary immobilization is recommended after reduction to maintain joint stability and reduce patient discomfort. In resource-limited environments, long leg splints or simple plaster casts may serve as viable options when braces are unavailable. Postreduction radiographs are essential to confirm realignment and identify associated fractures.^20^

External fixation is indicated in grossly unstable knees—particularly in the setting of vascular injury requiring revascularization, open dislocations, or notable soft-tissue trauma. It provides stability, allows wound access, and facilitates safe transport when transfer to a higher-level center is necessary. In low-resource settings, external fixators are often reusable and may be assembled with basic instrumentation. It is important to remove the external fixator within 4 weeks to minimize the risk of knee stiffness.^2,20,21^

The optimal timing of ligamentous reconstruction remains controversial, but in constrained environments, surgical planning must adapt to logistical realities. When feasible, early intevention within 10-14 days facilitates better identification of structures, allowing repairs and augmentations (repair combined with reconstruction). At this stage, capsular cicatrization has already occurred, making arthroscopy safer; nonetheless, arthroscopy should preferably be performed without continuous irrigation, with certain steps carried out as a dry procedure to reduce the risk of compartment syndrome. Delayed reconstruction—typically performed 3 to 6 weeks postinjury—is also an option, allowing time for soft-tissue recovery, completion of vascular repair, and availability of surgical resources. In selected cases, where surgical access is unpredictable or delayed treatment is not feasible, early definitive repair may be justified.^21-23^

Ultimately, stabilization strategies in low-income settings require balancing clinical urgency with system limitations. A staged approach—initial reduction and external fixation, followed by delayed ligamentous reconstruction—remains the most pragmatic pathway in most cases.

## Surgical Strategies in Low-income Environments

MLKIs, frequently associated with high-energy trauma, often require complex reconstructive procedures. In low-income settings, however, surgical strategies must be tailored to limited implant availability, reduced operating room time, and lack of advanced instrumentation. A growing body of literature supports pragmatic, evidence-based approaches using autografts, simplified fixation techniques, and reusable or implant-free solutions.^24,25^

### Use of Autografts

When allografts and advanced implants are unavailable, autografts remain the most feasible option. Hamstring tendons (semitendinosus and gracilis) are commonly harvested, but alternative graft sources such as the quadriceps tendon (QT), rectus femoris (RF), and peroneus longus have demonstrated adequate biomechanical properties.^26-28^

The QT graft is gaining popularity in multiligament knee reconstructions, especially for the anterior cruciate ligament (ACL),^29,30^ but it remains one of the least used autografts.^31^ Biomechanical studies indicate that the QT graft has superior structural properties compared with the bone-patellar tendon-bone (BPTB) graft,^32,33^ and a meta-analysis has shown functional outcomes comparable with those of hamstring and BPTB grafts.^34^ Furthermore, the QT is a versatile graft that can be harvested in different ways.^35,36^ Among these techniques, harvesting the superficial layer of the QT—known as the RF graft—has been gaining prominence.^37^

The peroneus longus tendon, in particular, has gained attention due to its sufficient length, easy harvest, and minimal donor site morbidity, especially in cases of hemitendon harvest, where it is split longitudinally and the anterior portion is used as a graft. Studies suggest that it may serve as a viable substitute for hamstring grafts in ACL and multiligament reconstructions.^38^

### Single Tunnel and Implantless Fixation Techniques

To reduce surgical time and material use, single femoral tunnel techniques have been developed for combined ligament reconstructions. For example, a shared tunnel can accommodate both posterior cruciate ligament (PCL) and medial collateral ligament (MCL) grafts, or ACL, lateral collateral ligament (LCL), and anterolateral ligament (ALL) structures, minimizing tunnel convergence and preserving bone stock.^2^

Implantless fixation techniques—such as press-fit fixation, bone bridges, and cortical suspensory methods using knotless suture loops—eliminate the need for expensive implant. These techniques, when executed correctly, have shown acceptable biomechanical strength in both cadaveric and clinical studies.^39,40^ Their use is especially valuable in systems without consistent implant supply or sterilization facilities.

### Reuse and Reprocessing of Surgical Materials

In some low-resource hospitals, sterilized and reused metallic screws and plates have been used because of cost constraints. While controversial, studies from trauma and reconstructive surgery have demonstrated that, with strict protocols for sterilization and visual inspection, reprocessed implants can be safely used in selected cases.^41,42^ Nonetheless, surgeons must weigh potential legal and infection risks, and this approach should follow institutional policies.

### Adapted Step-by-step Surgical Approach

A simplified, resource-conscious technique for acute MLKI reconstruction may include the following steps:

1. Exposure: A single longitudinal incision provides access to medial and central structures.
2. Graft harvest: Ipsilateral hamstrings are harvested. In their absence, peroneus longus or RF may be considered.
3. Tunnel preparation: One femoral tunnel is drilled for both cruciate and collateral structures if applicable.
4. Fixation: Use of press-fit graft ends or suturing over cortical bone bridges to avoid screws or buttons.
5. Staged procedures: When feasible, posterior and lateral structures can be reconstructed later, depending on patient stability, tissue condition, and surgical logistics. Some patients undergoing staged procedures decline ACL reconstruction after initial PCL and collateral ligament reconstruction, as they consider their knees satisfactory - particularly in the setting of partial ACL lesions.
6. This staged, modular approach offers surgeons flexibility while conserving resources and minimizing complications.

## Surgical Strategies: Injury Combinations

A consolidated summary across injury patterns is provided in Table 1.

**Table 1:** Decision Matrix for Multiligament Knee Injuries in Low-resource Settings

Injury Pattern	Recommended Grafts	Tunnel Strategy	Fixation Method	Suggested Technique (Citation)
ACL + PCL	Hamstrings, peroneus longus, rectus femoris, BPTB	Confluent or independent femoral tunnels	Press-fit, bone bridge, screws, staples	Confluent tunnel technique (Richter et al^2^)
ACL + ALL + PCL	Hamstrings, peroneus longus, rectus femoris	ACL + ALL shared femoral tunnel, PCL independent	Bone bridge, press-fit, cortical button	Single tunnel ACL + ALL (Barroso et al^37^)
ACL + LCL + PCL	Hamstrings, rectus femoris, peroneus longus	Independent tibial + femoral tunnels	Press-fit, screws, bone bridge	Adapted LaPrade with ACL anteriorized
ACL + PCL + PLC	Hamstrings, rectus femoris, peroneus longus	Independent femoral + fibular tunnels	Press-fit, bone bridge, screws	Modified LaPrade or Franciozi technique
ACL + LCL + MCL	Hamstrings, fascia lata, semitendinosus	Three femoral tunnels, shared tibial tunnel	Press-fit, screws, bone bridge	One-stage ACL-MCL (Gobbi et al^45^)
ACL + MCL	Hamstrings, rectus femoris, fascia lata	Independent femoral tunnels	Press-fit, screws, bone bridge	Canuto or Franciozi MCL technique
PCL + MCL	Hamstrings, rectus femoris, peroneus longus	Independent femoral tunnels	Press-fit, screws, bone bridge	Bonadio or Franciozi technique
ACL + PLC	Rectus femoris, peroneus longus, BPTB	Shared femoral tunnel (ACL + PLC), fibular tunnel for PLC	Press-fit, screws, staples	Shared tunnel ACL + PLC (Melo et al^58^)
MCL + LCL	Semitendinosus, peroneus longus, fascia lata	Femoral + tibial (MCL), femoral + fibular (LCL)	Bone bridge, screws, staples	Triangular sling + LCL reconstruction
ACL + PCL + MCL + LCL	Hamstrings, rectus femoris, peroneus longus	Staggered femoral tunnels	Press-fit, bone bridge, screws, staples	Staged MLKI (Schenck et al^7^)

ACL = anterior cruciate ligament, ALL = anterolateral ligament, BPTB = bone-patellar tendon-bone, LCL = lateral collateral ligament, MCL = medial collateral ligament, MLKI = multiligament knee injury, PLC = posterolateral corner, PCL = posterior cruciate ligament

Notes: Techniques referenced correspond to items cited in the manuscript reference list; no additional references are introduced within the table.

Summary of injury patterns with recommended autografts, tunnel strategies, fixation methods, and technique references tailored to constrained environments.

### Anterior Cruciate Ligament + Posterior Cruciate Ligament Injuries

Graft choices: Ipsilateral hamstring tendons (semitendinosus and gracilis) are commonly used due to availability and minimal donor site morbidity. The peroneus longus tendon and RF are a viable alternative, especially when larger grafts or longer constructs are needed. BPTB is an option that should always be considered for ACL reconstruction.^43^

Tunnel configuration: Single-bundle reconstructions for both ACL and PCL are recommended in low-resource settings. A shared femoral tunnel, known as the “confluent tunnel” approach, reduces surgical time, avoids tunnel convergence, and minimizes implant requirements.^2,44^

#### Fixation Options

Implantless techniques: Press-fit femoral fixation and tibial bone bridges can be used when implants are unavailable. These techniques use graft compression against cancellous bone or cortical ridges for mechanical stability.^45-47^

Low-cost implant techniques: When basic implants are available, the use of a metallic interference screw on the femoral side and a surgical staple (agrafe) on the tibial cortex offers an effective, reproducible, and affordable alternative.^48,49^

### Anterior Cruciate Ligament + Anterolateral Ligament + Posterior Cruciate Ligament Injuries

Graft choices: Given the complexity of this combined pattern, thoughtful graft allocation is critical in low-resource settings. Three clinically feasible configurations include

Option 1: Semitendinosus and gracilis tendons (hamstring) or BPTB for ACL, hemiperoneus longus tendon graft for ALL, and RF tendon for PCL reconstruction.^50^

Option 2: RF used as a double graft for both ACL and ALL (when of adequate length >24 cm), hamstring tendons for PCL.^37^

Option 3: Tripled hamstring graft (semitendinosus + gracilis) in braid configuration for ACL,^51^ gracilis for ALL, and RF for PCL.

Option 4: Semitendinosus and gracilis tendons (hamstring) for ACL, lemaire tenodesis for ALL, and RF tendon for PCL reconstruction.^52^

#### Tunnel Configuration

A single femoral tunnel for both ACL and ALL can be used, with the graft limbs diverging toward the tibial ACL and ALL insertion sites. This minimizes tunnel convergence and reduces surgical complexity. PCL femoral and tibial tunnels remain independent but can follow simplified trajectories.

#### Fixation

Implantless: Bone bridge on the tibial side (ACL), press-fit on the femoral side, and ALL may be sutured directly to periosteum.

#### Low-cost Implant Technique

A metallic interference screw on the femoral ACL/PCL tunnel, staple (agrafe) for tibial ACL or ALL insertions, suspensory cortical button if available for ACL and ALL on femoral fixation, and BPTB press-fit fixation can be recommended as a fixation technique for ACL reconstruction.

### Anterior Cruciate Ligament + Lateral Collateral Ligament + Posterior Cruciate Ligament Injuries

#### Graft Choices

Option 1: Hamstring or BPTB for ACL, hemiperoneus longus tendon graft for LCL, and RF tendon for PCL reconstruction.^50^

Option 2: RF used as a double graft for both ACL and LCL (when of adequate length >24 cm) and hamstring tendons for PCL.^37^

Tunnel configuration: Independent tibial and femoral tunnels. To reduce tunnel crowding, position ACL tunnel more anteriorly.

#### Fixation Options

Press-fit graft ends into femoral sockets

Bone bridge tibial anchoring or suture tying over cortical buttons (metal or reused implants)

Interference screws (metallic, nonbiodegradable) in femur and tibia

Staples (agrafes) for LCL or PCL tibial fixation

Suspensory buttons or washers repurposed from trauma sets if available

BPTB press-fit fixation can be recommended as a fixation technique for ACL reconstruction.

#### Surgical Technique

A simplified version of the LaPrade anatomic posterolateral corner (PLC) reconstruction can be adapted using a single long autograft passed through the fibular head and fixed in separate femoral tunnels for the LCL and popliteus.^53^ This avoids conflict with the ACL tunnel and reduces surgical steps. The ACL can be reconstructed in a standard outside-in fashion and fixed with press-fit, screw, or suture-loop technique depending on graft and resources.

Timing: In settings with limited access to ORs, single-stage reconstruction is recommended.

### Anterior Cruciate Ligament + Posterior Cruciate Ligament + Posterolateral Corner Injuries

This combination is one of the most severe patterns of knee dislocation, resulting in global instability with anterior, posterior, and posterolateral laxity. Management requires coordinated reconstruction of the central axis (ACL and PCL) and the PLC, which, if unaddressed, leads to high failure rates of cruciate reconstructions. Although closely related within the lateral knee anatomy, injuries to the PLC and the LCL are not the same. The LCL is one of the key static stabilizers of the lateral compartment, resisting varus stress. By contrast, the PLC is a broader anatomic complex that includes the LCL, popliteus tendon, and popliteofibular ligament, among other structures. Although an isolated LCL injury results in linear varus instability, a true PLC injury causes combined varus and external rotational instability. Failure to identify and treat a complete PLC injury—beyond just the LCL—can lead to reconstruction failure of both ACL and PCL.

Preferred Graft Choices in Low-resource Settings

#### Preferred Strategy

Hamstring tendons for ACL or PLC, QT, or RF tendon autograft: Preferred for either ACL or PCL due to its robustness, length, and preservation of hamstrings.

Hemiperoneal autograft: (Split peroneus longus) ideal for popliteus or PFL components of PLC.

BPTB: When feasible, especially for ACL, allows press-fit fixation without implants.

#### Alternative Options

Biceps femoris tendon (split): Can be used for LCL reconstruction, but may cause lateral instability or morbidity in already damaged PLCs.

Plantaris tendon: Suitable for short graft needs (e.g., PFL), but variable in anatomy and diameter.

#### Tunnel Configuration

ACL and PCL: Independent tunnels for tibial and femoral fixation. Careful angulation to avoid tunnel convergence with PLC.

#### Posterolateral Corner

Femoral tunnel shared for LCL and popliteus

Fibular tunnel for PFL

Avoid tibial PLC tunnels in low-resource environments.

Fixation methods—Implantless/low-cost:

Press-fit fixation in femoral and tibial sockets with BPTB

Bone bridges on tibia or fibula

Sutures over cortical bone for PFL or LCL anchoring.

#### Metallic Implant

Interference screws (nonbiodegradable) for femoral ACL/PCL tunnels

Surgical staples (agrafes) for securing tendons at tibial/fibular ends

Reused trauma implant (e.g., washers and buttons).

#### Surgical Technique Options

1. Anatomic PLC Reconstruction with Autografts—Franciozi et al.^54^
  Describes the use of three autografts (gracilis, semitendinosus, and biceps femoris) to reconstruct LCL, popliteus, and PFL in their respective tunnels. Adaptable with
  Press-fit or metallic screw fixation
  Compatible with RF or hemiperoneal substitutions
  Step-by-step guidance for tunnel creation and graft passage.
2. Modified LaPrade Technique—Single-graft Loop
  Using a long autograft passed through the fibular tunnel and fixed into two femoral sockets for LCL and popliteus. Minimizes graft harvesting.^55^
3. Outside-in ACL + PCL Reconstruction + Staged PLC
  Ideal for settings where full reconstruction must be staged. Begin with ACL + PCL using BPTB or RF with press-fit. Add PLC later with hamstrings or peroneus longus.^2^
  Timing: In settings with limited access to ORs, single-stage reconstruction is recommended.

### Anterior Cruciate Ligament + Lateral Collateral Ligament + Medial Collateral Ligament Injuries

This tricompartmental injury presents with global coronal instability. While uncommon, it may result from high-energy varus-valgus stress combined with ACL rupture (e.g., motorcycle trauma and sports with twisting impacts). Simultaneous reconstruction of both medial and lateral complexes is technically demanding and must be carefully planned to avoid tunnel convergence and soft-tissue crowding.

#### Graft Options

ACL: Hamstring or BPTB or RF tendon

LCL: Peroneus longus tendon

MCL: Semitendinosus tendon with tibial tunnel, as described by Canuto et al.^56^

Alternative: Tibial sling with triangular configuration, including a posterior oblique ligament (POL) limb^57^

Others: Fascia lata, QT (when hamstrings are preserved).

#### Tunnel Strategy

ACL: Independent femoral and tibial tunnels

LCL: Femoral and fibular tunnels (PLC must be ruled out before considering isolated LCL)

MCL: Canuto technique: Femoral tunnel + two tibial tunnels (anterior and posterior to pes anserinus). Franciozi technique: Tibial sling from femoral isometric point to a triangular fixation point on tibia + POL reinforcement.

Care must be taken to stagger tunnels and avoid convergence between MCL and ACL on the medial femoral condyle.

#### Fixation Techniques

Implantless: Press-fit femoral fixation for ACL, bone bridge, or suture loop for tibial MCL attachment

Implants: Metallic screws (nonbiodegradable) and staples (agrafe) for MCL tibial exit

Suspensory fixation for ACL or MCL when adequate soft-tissue coverage is present.

#### Surgical Strategy

Stage 1: ACL + MCL reconstruction if lateral side is stable or braceable

Stage 2: LCL or PLC reconstruction after tissue recovery.

In unstable cases or poor follow-up environments, perform all reconstructions in one stage.

### Anterior Cruciate Ligament + Medial Collateral Ligament Injuries

This combination is relatively more common, especially in valgus-external rotation injuries or contact sports. MCL injuries are often managed nonoperatively; however, high-grade tears (grade III), or persistent instability after ACL reconstruction, require surgical treatment.

ACL: Hamstring or BPTB or RF tendon

MCL: Semitendinosus tendon with tibial tunnel, as described by Canuto et al.^56^

Alternative: Tibial sling with triangular configuration^57^

Others: Fascia lata, QT (when hamstrings are preserved), and peroneus longus tendon.

#### Tunnel and Fixation Strategy

ACL: Independent femoral and tibial tunnels

MCL: Femoral tunnel at isometric point (posterior and proximal to medial epicondyle). Canuto^56^: Or two tibial tunnels with sutured graft ends.^57^

#### Fixation

Bone bridge and cortical suture loops

Screws and staples when available

#### Timing and Strategy

In isolated ACL + MCL lesions:

Grade I–II MCL: Treat nonoperatively, reconstruct only ACL

Grade III MCL: Reconstruct both, ideally single-stage reconstruction is recommended.

### Posterior Cruciate Ligament + Medial Collateral Ligament Injuries

Injuries involving the PCL and medial collateral ligament often result from direct anterior trauma to the proximal tibia with valgus force, such as dashboard injuries in vehicular accidents or sports tackles. These lesions cause posterior tibial translation associated with valgus instability, especially near full extension. When untreated, they lead to chronic instability and medial joint overload.

Although isolated MCL injuries are often treated nonoperatively, grade III tears associated with PCL ruptures generally require surgical reconstruction to restore both sagittal and coronal stability.

#### Graft Strategy

In low-resource settings, optimal graft choices should prioritize availability, biomechanical sufficiency, and ease of harvest:

PCL: Hamstring or RF tendon or peroneus longus

MCL: Femoral tunnel at isometric point (posterior and proximal to medial epicondyle). Canuto^56^: Or two tibial tunnels with sutured graft ends.^57^

#### Tunnel Configuration

PCL: Independent femoral and tibial tunnels

Tibial tunnel drilled using the anteromedial approach with posterior protection

Femoral socket angled anteroproximally to avoid overlap with the MCL tunnel

MCL: Canuto technique^56^: One femoral tunnel at isometric point (posterior and proximal to medial epicondyle). Two tibial tunnels, anterior and posterior to pes anserinus. Franciozi technique^57^: One tibial sling tunnel, single femoral tunnel + limb fixed posteriorly for POL.

Fixation Options/Low-cost fixation strategies:

PCL: Metallic screw in femur; agrafe or suture loop in tibia

MCL: Bone bridge or cortical tie-over at tibia

Femoral press-fit or screw

Suture anchors or staples (if available) at tibial side for sling/POL.

Surgical Strategy

In isolated ACL + MCL lesions:

Grade I–II MCL: Treat nonoperatively, reconstruct only PCL if necessary

Grade I–II PCL: Treat nonoperatively, reconstruct only MCL if necessary

Grade III MCL and PCL: Reconstruct both, ideally single-stage reconstruction is recommended.

### Anterior Cruciate Ligament + Posterolateral Corner Injuries

This combination involves anterior and posterolateral instability, often resulting from high-energy trauma or overlooked posterolateral injury during ACL reconstruction. Isolated ACL reconstruction in the presence of unrecognized PLC injury frequently results in persistent instability, graft elongation, and ultimate failure. Correcting both structures—especially in a coordinated, anatomically accurate, and low-cost fashion—is essential.

#### Preferred Strategy

Hamstring tendons: For either ACL or PLC

Hemiperoneal tendon: Suitable for PFL or popliteus components of PLC

BPTB: When available, ideal for ACL with press-fit bone-to-bone fixation

RF tendon autograft: Robust and long, ideal for ACL or PLC reconstruction using loop or sling techniques.

#### Tunnel Configuration

ACL: Tibial and femoral tunnels as per standard techniques. Or shared femoral tunnel with PLC possible, as described by Melo et al.^58^

PLC: Popliteus implant passed through the same femoral tunnel as the ACL. Fibular tunnel used for popliteofibular ligament (PFL) limb. Avoid tibial PLC tunnels in low-resource environments to minimize risk and cost.

Fixation Methods—Implantless/Low-cost Options.

Femoral socket:

Press-fit fixation for both ACL and popliteus graft limbs (ideal for BPTB).

#### Shared tunnel reduces implant use

Tibial/Fibular side:

Bone bridge or periosteal suture for PFL graft

Staples (agrafe) or reused trauma washers for securing grafts.

#### Surgical Technique Options

1. Shared Femoral Tunnel Technique—Melo et al.^58^
  Describes a revision ACL + PLC reconstruction using a single femoral tunnel for both structures.
  Grafts diverge from the femur toward the tibial and fibular fixation points.
  Ideal for low-resource or revision settings where femoral bone stock is limited.
  Compatible with RF, semitendinosus, or BPTB grafts.
  Fixation options include press-fit, screws, or low-cost staples.
2. Anatomic PLC Reconstruction with Autografts—Franciozi et al.^54^
  Uses three autografts (gracilis, semitendinosus, and biceps femoris) for LCL, popliteus, and PFL.
  Requires independent tunnels; may be adapted to shared or simplified constructs.
3. Modified LaPrade Technique—Single-graft Loop^59^
  A single long graft passed through fibular tunnel and fixed into femoral sockets for LCL and popliteus.
  Reduces graft harvest and allows staged addition of PCL or ALL if needed.

#### Timing

In scenarios with predictable odds ratio access and stable soft tissues, single-stage reconstruction is preferred. However, in acute trauma or when tissue status is poor:

Stage 1: ACL with bracing treatment
Stage 2 (2 to 6 weeks): PLC reconstruction, using shared tunnel if feasible.

### Medial Collateral Ligament + Lateral Collateral Ligament Injuries

Simultaneous injuries of the medial and LCLs (MCL + LCL) without cruciate involvement are rare but biomechanically significant, often resulting from direct varus-valgus forces applied in full extension. These injuries may present with gross coronal plane instability and often require surgical repair or reconstruction, especially when grade III.

#### Graft Strategy

In low-resource settings, autografts are prioritized for cost-effectiveness and availability:

MCL: Preferred: Semitendinosus tendon using the Canuto technique^56^ (femoral + two tibial tunnels). Alternative: Franciozi^57^ triangular sling technique for additional posteromedial reinforcement.

LCL: Preferred: Peroneus longus. Alternative: Fascia lata (when lateral tissue is compromised) or biceps femoris slip.

#### Tunnel Strategy

MCL: Femoral isometric tunnel + two tibial tunnels anterior and posterior to the pes anserinus or single tibial sling tunnel

LCL: Independent femoral tunnel and fibular tunnel.

Tunnel spacing must be carefully planned to avoid convergence on the femoral condyle and allow strong, isometric fixation.

#### Fixation Method

Implantless: Bone bridge, press-fit, and periosteal suture fixation.

#### Implants

Screws (metallic and reusable)

Surgical staples

Suspensory loops for LCL femoral tunnel, if available.

#### Surgical Strategy

In isolated MCL + LCL injuries, single-stage reconstruction is feasible and encouraged.

Grade I–II MCL: Treat nonoperatively, reconstruct only LCL if necessary

Grade III MCL: Reconstruct both, ideally single-stage reconstruction is recommended.

### Anterior Cruciate Ligament + Posterior Cruciate Ligament + Medial Collateral Ligament + Lateral Collateral Ligament (Complete Multiligament Knee Injury)

Complete dislocation involving rupture of all four primary ligaments (ACL, PCL, MCL, and LCL) represents the most severe form of MLKI. These injuries are commonly due to high-energy trauma, often with neurovascular compromise, and require carefully staged, resource-optimized reconstruction to preserve joint function and limb viability.

#### Graft Strategy

Given the extent of reconstruction, careful planning of graft allocation is essential:

ACL: BPTB, hamstrings, or RF

PCL: Peroneus longus, RF, or hamstrings (tripled if necessary)

MCL: Semitendinosus using Canuto technique or Franciozi sling

LCL: Peroneus longus or biceps femoris slip.

In many cases, bilateral graft harvest (contralateral hamstrings or rectus) may be necessary due to autograft limitations on the injured side.

#### Tunnel Strategy

Use staggered femoral tunnels to avoid convergence

Share tunnels when possible (eg, ACL + LCL through extended trajectory)

Prioritize ACL/PCL central axis first, then reconstruct collateral ligaments.

#### Fixation Method

Implantless: Press-fit femoral/tibial fixation, cortical suture loops, and bone bridges.

Implant options (as available):

Interference screws

Surgical staples

Suspensory devices or reused trauma implants.

Surgical Strategy: Staged Modular Approach (Schenck & Richter)^7,9^

Stage 1:

Joint reduction, external fixation if grossly unstable

Vascular repair (if needed)

Initial central axis reconstruction (PCL and/or ACL).

Stage 2 (3 to 6 weeks):

MCL and LCL reconstruction, once soft tissue envelope stabilizes

Use remaining autografts or fascia lata if needed.

In single-stage cases (stable skin/soft tissues, odds ratio access available), all structures may be addressed together using modular tunnel strategy.

## Rehabilitation and Follow-up in Vulnerable Populations

Rehabilitation is a critical determinant of functional outcome after MLKI, yet it remains a major challenge in low-income settings. Limited access to formal physical therapy, geographic barriers, and socioeconomic constraints frequently compromise adherence to structured postoperative protocols.^5^

### Simplified Rehabilitation Protocols

In resource-constrained environments, rehabilitation protocols must be both effective and feasible. Key principles include early mobilization to prevent stiffness, protection of reconstructed structures, and progressive strengthening.^60^ A practical low-cost protocol may include

Weeks 0 to 2: Immobilization in extension using a long leg brace or posterior splint, isometric quadriceps activation, and ankle pumps.

Weeks 2 to 6: Initiation of passive and active-assisted range of motion exercises, non–weight-bearing or partial weight-bearing with crutches depending on surgical stability. If the PCL has been reconstructed, passive motion is recommended to be performed in the supine position.

Weeks 6 to 12: Transition to full weight-bearing, closed kinetic chain exercises, and stationary cycling when available. Stationary cycling should be introduced if the PCL has not been reconstructed.

Months 3 to 6: Functional training including balance and proprioception, gradual return to work-related tasks.

After 6 months: Clearance for sport-specific drills in patients with high-demand profiles, depending on surgical success and limb function. However, patients undergoing multiligament reconstruction typically return to sports no earlier than one year after the initial reconstruction.

This protocol prioritizes home-based rehabilitation and minimizes dependence on in-person physical therapy visits.^5^

### Strategies to Ensure Adherence

To improve compliance in vulnerable populations, several community-based strategies have proven effective:

Printed or video-based home exercise guides, culturally adapted and pictorially illustrated.

Involvement of family members or community health agents to assist with exercise supervision.

Group rehabilitation sessions organized at local health centers on weekly or biweekly schedules.

Use of mobile health technologies, such as text reminders or WhatsApp video check-ins, when feasible.^61^

Patients should be educated early about the importance of movement and adherence, ideally during the initial hospitalization period.^61^

### Criteria for Manipulation Under Anesthesia

Postoperative stiffness is a common complication, especially in delayed or inadequately supervised rehabilitation. Manipulation under anesthesia (MUA) may be considered in patients who, by 6 to 8 weeks postoperatively, present with^60^ < 90° of knee flexion, despite adherence to passive mobilization, fixed extension loss >10°, and limiting gait or brace use.

Pain-free joint with no mechanical block, confirmed by clinical examination.

In low-income settings, MUA can be done under spinal or regional anesthesia with minimal instrumentation. However, efforts should be made to avoid stiffness through early range of motion exercises and routine follow-up.^60^

## Barriers and Challenges in Resource-constrained Contexts

The management of knee dislocations in low-income settings is fraught with systemic barriers that extend beyond the surgical technique. Understanding these challenges is essential for implementing realistic, context-sensitive solutions.^2^

### Logistical and Infrastructure Limitations

Patients in underserved regions often face delays in diagnosis and treatment due to poor access to emergency care, lack of trauma systems, and geographic isolation.^2^ Many health facilities are not equipped with advanced imaging modalities, and referral networks are weak or inconsistent. In addition, surgical capacity is limited by low operating room availability, lack of implants, and insufficient sterilization infrastructure.

Postoperative rehabilitation is also compromised. Access to physical therapy is often restricted to urban centers, leaving rural patients without follow-up support. Equipment such as continuous passive motion machines, hinged braces, or resistance tools is rarely available, and most patients cannot afford to purchase them independently.^5^

### Cultural, Economic, and Social Constraints

Cultural beliefs about immobility, pain, and surgery may affect patient compliance with treatment and rehabilitation.^3^ Some communities prioritize rapid return to work over long-term recovery, leading to early discontinuation of bracing treatment or therapy. Literacy and language barriers can hinder communication of instructions, especially for home-based rehabilitation protocols.^5^

Economically, many patients lack health insurance or social support systems. Transportation costs to attend follow-up appointments may be prohibitive, and out-of-pocket payments for implants or medications limit access to adequate care.^5^

### Sustainable Improvement Proposals

Improving outcomes in these environments requires low-cost, scalable interventions tailored to local realities. Suggested strategies include

1. Decentralized surgical education, training general orthopaedic surgeons in trauma centers to perform essential MLKI reconstructions with autografts and minimal implants.^5^
2. Development of low-cost surgical kits, including reusable external fixators and autograft instrumentation.^42^
3. Community-based rehabilitation models, involving local health workers to support recovery through home visits and structured group sessions.^5^
4. Creation of simplified treatment algorithms, guiding decision-making when access to vascular or neurosurgical backup is limited.^17^
5. Implementation of mobile health (mHealth) platforms, such as WhatsApp-based follow-up, to overcome geographic barriers.^61^
6. Partnerships with NGOs and university hospitals, to establish donation programs for surgical supplies and create sustainable supply chains for implants.^2^

By addressing these multifactorial challenges through structured yet adaptable interventions, it is possible to improve the standard of care for knee dislocation patients—even in the most constrained environments.

### Synthesis of the Evidence and Practical Guidelines

Despite the complexity of MLKIs and the structural limitations faced in low-income settings, recent literature demonstrates that acceptable outcomes can be achieved with adapted, evidence-informed strategies. This review highlights practical, resource-conscious approaches that prioritize early recognition, simplified diagnostics, judicious use of autografts, and staged surgical care.

## Phase-based Clinical Management

To guide orthopaedic teams operating under constraints, we propose a phase-based management framework:

### Acute Phase (0 to 2 weeks)

Goal: Limb salvage, early diagnosis, pain control.

1. Immediate closed reduction and neurovascular assessment
2. Use of ABI and Doppler when CTA is unavailable
3. Temporary immobilization with long leg splint or cast
4. External fixation in open or unstable knees
5. Urgent referral for confirmed vascular injury
6. Early patient education regarding risks of stiffness and instability.

### Subacute Phase (2 to 6 weeks)

Goal: Stabilization and planning of definitive treatment

1. Delay ligament reconstruction until soft tissue conditions improve
2. Monitor for compartment syndrome and ROM restrictions
3. Prioritize staged surgery if instability compromises function
4. Harvest autografts (hamstrings, BPTB, QT, peroneus longus, and RF)
5. Reuse of sterilized materials may be considered under the strict protocol [13].

### Chronic Phase (>6 weeks)

Goal: Restore joint stability and function.

1. Perform staged reconstructions: Central axis first (ACL/PCL), then peripheral
2. Prefer implantless fixation (press-fit, bone bridge) when cost limits implant use
3. Simplified rehabilitation protocol with home-based guidance
4. Manipulation under anesthesia if flexion <90° at 6 to 8 weeks
5. Community-based follow-up or mHealth strategies improve adherence.

## Practical Algorithm for Knee Dislocation in Low-resource Settings

Step 1: Suspicion of dislocation →

1. Clinical examination, reduce urgently if dislocated
2. Document vascular status before and after reduction.

Step 2: Vascular evaluation →

1. ABI <0.9 or abnormal Doppler → CTA or surgical exploration
2. Vascular repair → external fixation + staged ligament surgery.

Step 3: Ligament assessment →

1. Classify injury pattern using Schenck system
2. Consider plain radiographs ± MRI if available.

Step 4: Surgical strategy →

1. Early repair if tissues permit; otherwise delay 2 to 6 weeks
2. Use autografts, avoid implants when possible.

Step 5: Rehabilitation →

1. Simplified protocol with local health worker supervision
2. Consider MUA at 6 to 8 weeks if ROM <90°.

## Conclusion

Knee dislocations are complex, high-risk injuries that demand timely recognition, structured evaluation, and coordinated management. In resource-constrained environments, these challenges are compounded by limited access to diagnostics, surgical implants, specialized rehabilitation, and follow-up care. Despite these barriers, this review demonstrates that effective, evidence-based care is possible through adaptation, prioritization, and innovation.

Key strategies include the use of autografts such as hamstrings, RF, and peroneus longus; implantless fixation techniques including press-fit and bone bridges; and simplified rehabilitation protocols with community support. A phase-based approach—spanning acute reduction, subacute planning, and staged reconstruction—provides a pragmatic framework for clinical decision-making in underserved regions. In addition, low-cost educational and logistical interventions can improve treatment adherence and outcomes.

From a clinical standpoint, this review offers practical guidelines that can be immediately implemented by orthopaedic surgeons working in low-income or remote areas. From a scientific perspective, it underscores the urgent need for high-quality studies on surgical outcomes, rehabilitation strategies, and system-level innovations in low-resource trauma care.

Future research should focus on validating simplified surgical techniques, evaluating community-based rehabilitation models, and developing low-cost surgical kits specifically designed for MLKIs. Ultimately, improving care for knee dislocation in low-income settings requires both clinical creativity and structural reform—guided by global equity in musculoskeletal health.
